# Aktueller Stand der rhythmologischen Ausbildung in Deutschland

**DOI:** 10.1007/s00399-020-00717-4

**Published:** 2020-09-01

**Authors:** Kevin Willy, Patrick Müller, Lars Eckardt, David Duncker

**Affiliations:** 1 Klinik für Kardiologie II – Rhythmologie, Westfälische Wilhelms-Universität Münster, 48149 Münster, Deutschland; 2 Hannover Herzrhythmus Centrum, Klinik für Kardiologie und Angiologie, Medizinische Hochschule Hannover, Hannover, Deutschland

**Keywords:** Facharztausbildung, Kardiologische Weiterbildung, Rhythmologische Ausbildung, Fortbildung, Specialist training, Cardiovascular training, Medical training, Fellowship

## Abstract

**Hintergrund:**

Die rhythmologische Ausbildung junger Kardiologinnen und Kardiologen bildet das Fundament für eine optimale Versorgung von Patienten mit Herzrhythmusstörungen. Die Rhythmologie zeichnet sich durch einen hohen Innovationsgrad aus und hat sich innerhalb der letzten Jahre rasant weiterentwickelt. Dies könnte zu einer Kluft zwischen der klinischen Ausbildung und neuer Technologien geführt haben und die Ausbildungsanforderungen maßgeblich verändern.

**Fragestellung:**

Das Ziel der Umfrage war es, ein Meinungsbild junger Kardiologinnen und Kardiologen über den Stellenwert und die Zufriedenheit der rhythmologischen Ausbildung zu erheben und Verbesserungsansätze zu formulieren.

**Methoden:**

Die Umfrage wurde im Mai 2020 durchgeführt. Mitglieder der Sektion Young-DGK der deutschen Gesellschaft für Kardiologie wurden via E‑Mail kontaktiert und gebeten einen onlinebasierten Fragebogen zu beantworten. Der Fragebogen beinhaltete Informationen über den aktuellen Stellenwert, die Zufriedenheit und die Strukturen der rhythmologischen Ausbildung in Deutschland.

**Ergebnisse:**

Insgesamt 131 Young DGK-Mitglieder (68 % männlich) nahmen an der Umfrage teil. Das Durchschnittsalter betrug 33 ± 3,3 Jahre und der Ausbildungsstand gliederte sich wie folgt: 64 % Assistenzärzte, 20 % Fachärzte und 16 % Oberärzte. 72 Teilnehmer (53 %) berichteten, mit ihrer kardiologischen Weiterbildung sehr zufrieden zu sein. Die Hälfte der Teilnehmer wünschte sich einen höheren Umfang an rhythmologischen Inhalten in ihrer Weiterbildung. Rhythmologische Fortbildungsveranstaltungen und Fellowships wurden überwiegend positiv bewertet (70 % bzw. 93 %), jedoch waren diese jeweils für knapp die Hälfte der Teilnehmer unbekannt. Verbesserungsansätze sahen die Teilnehmer in der Einführung einer generellen rhythmologischen Rotation, einem gesteigerten Zugang zu Prozeduren um invasive Fähigkeiten zu erlernen (falls nötig auch klinikübergreifend), einer intensivierten Werbung für Fortbildungsveranstaltungen und Fellowships sowie einer Zunahme onlinebasierter Fortbildungsveranstaltungen.

**Schlussfolgerung:**

Die Umfrage unterstreicht das Interesse junger Kardiologinnen und Kardiologen an einer strukturierten und intensivierten rhythmologischen Ausbildung innerhalb ihrer kardiologischen Weiterbildung.

**Zusatzmaterial online:**

Zusätzliche Informationen sind in der Online-Version dieses Artikels (10.1007/s00399-020-00717-4) enthalten.

## Abstract (en)

**Background:**

Cardiac electrophysiology training of young cardiologists forms the foundation of optimal heart rhythm patient care. In particular, cardiac electrophysiology is characterized by continuous innovation and has progressed rapidly over the last few years. This may have led to a gap between clinical training and the new technologies and could significantly change training requirements.

**Objective:**

The aim of the survey was to obtain the opinion of young cardiologists about the current importance and satisfaction of cardiac electrophysiology training and to identify areas for improvement.

**Methods:**

The survey was conducted in May 2020. Young DGK (Deutschen Gesellschaft für Kardiologie, German Society for Cardiology) members were contacted via e‑mail and asked to answer an online-based questionnaire. The questionnaire containing information about the current status, satisfaction, and structures of educational and clinical proficiency of cardiac electrophysiology in Germany.

**Results:**

Overall, 131 young DGK members completed the survey. Mean age was 33 ± 3.3 years and 68% of the participants were male. Among the participants, 64% were cardiology fellows, while 36% were qualified. A total of 72 participants (53%) reported overall satisfaction with their clinical training and 50% of the participants held the opinion that cardiac electrophysiology should become a larger part of their education. Training courses and fellowships were rated mostly positive (70% vs. 93%, respectively); however, the later were unknown to 41% of the participants. Areas identified for improvement included the following: establishment of general cardiac electrophysiology rotation; enabling access to procedures to gain invasive skills; increased advertising for training courses and fellowships; increase of online-based training courses.

**Conclusion:**

The survey underlines the interest of young cardiologists in an intensified cardiac electrophysiology training within their cardiology training.

Die rhythmologische Ausbildung junger Kardiologinnen und Kardiologen bildet das Fundament für eine optimale Versorgung von Patienten mit Herzrhythmusstörungen. Durch einen Wissenszuwachs und eine kontinuierliche technische Innovation in der kardiovaskulären Medizin sind junge Kardiologinnen und Kardiologen in ihrer beruflichen Laufbahn mit sich dynamisch entwickelnden diagnostischen und therapeutischen Optionen konfrontiert [1–3]. Insbesondere in der rhythmologischen Ausbildung sind die Voraussetzungen und zu erwerbenden Fähigkeiten innerhalb der letzten Jahre deutlich angestiegen [4]. Dies könnte zu einer Kluft zwischen der klinischen Ausbildung und neuer Technologien geführt haben und die Ausbildungsanforderungen maßgeblich verändern.

Die Arbeitsgruppe Elektrophysiologie und Rhythmologie (AGEP) der Deutschen Gesellschaft für Kardiologie (DGK), die DGK Akademie für Aus‑, Weiter- und Fortbildung, die europäische Gesellschaft für Kardiologie (ESC) sowie firmenbasierte Fellowships engagieren sich in der rhythmologischen Aus- und Weiterbildung durch ein großes Portfolio an Präsenz- und Online-Fortbildungsveranstaltungen. Die Young DGK ist eine Sektion der DGK zur Netzwerkbildung junger Mitglieder innerhalb der DGK, die einen ihrer Schwerpunkte in der Gestaltung der zukünftigen kardiologischen Aus- und Weiterbildung sieht.

Das Ziel der vorliegenden Umfrage war es, ein Meinungsbild von Young DGK-Mitgliedern über die Bedeutung der Rhythmologie und deren Ausbildungsstrukturen innerhalb ihrer kardiologischen Weiterbildung zu erheben und Verbesserungsansätze zu formulieren.

## Methoden

Die Umfrage wurde im Mai 2020 durchgeführt. Alle Young DGK-Mitglieder wurden via E‑Mail kontaktiert und gebeten, einen onlinebasierten Fragebogen über die Plattform SurveyMonkey (www.surveymonkey.com; SVMK Inc., San Mateo, CA, USA) zu beantworten. Die Umfrage bestand aus insgesamt 22 Fragen, die folgende Aspekte beinhaltete: demographische Daten, Informationen zum Ausbildungszentrum, Zufriedenheit mit der Aus- und Weiterbildung, Bewertung der Ausbildungsstrukturen, Wahrnehmung von Fortbildungsveranstaltungen und Fellowships und Verbesserungsansätze. Berechnungen zur deskriptiven Statistik wurden mit SPSS Version 24.0 durchgeführt.

## Ergebnisse

Insgesamt nahmen 131 Young DGK-Mitglieder an der Umfrage teil (Tab. 1); 89 Teilnehmer (68 %) waren männlich. Das Durchschnittsalter lag bei 33 ± 3 Jahren mit einer Altersspanne von 27 bis 43 Jahren. Der Ausbildungsstand der Teilnehmer gliederte sich wie folgt: 84 Assistenzärzte, 26 Fachärzte und 21 Oberärzte.

**Table 1:** 

**Demografie**	**(*n*** **=** **131)**
*Alter (Jahre)*	33,0 ± 3,3 (min. 27; max. 43)
*Geschlecht*	
Weiblich	42 (32 %)
Männlich	89 (68 %)
*Ausbildungsstand*	
Assistenzarzt	84 (64 %)
Facharzt	26 (20 %)
Oberarzt	21 (16 %)
**Arbeitsumfeld**	
*Arbeitsplatz*	
Universitätsklinik	71 %
Öffentliche Trägerschaft	11 %
Kirchliche Trägerschaft	7 %
Private Trägerschaft	11 %
*Eigenständige rhythmologische Abteilung*	88 (67 %)
*Leistungsspektrum*	
Invasive Elektrophysiologie	121 (92 %)
Operative Devicetherapie	123 (94 %)
**Bekanntheitsgrad von Fortbildungsveranstaltungen**	
*DGK-Sachkundekurse*	100 (76 %)
*Fellowship-Programme*	68 (52 %)
„ESCeL“ der ESC* (e-learning)*	64 (49 %)

*DGK* Deutsche Gesellschaft für Kardiologie,*ESC* European Society of Cardiology

Der Großteil der Teilnehmer war in einem Universitätsklinikum beschäftigt (71 %), während 11 % in einem öffentlichen, 11 % in einem privaten und 7 % in einem kirchlichen Krankenhaus tätig waren. Bei zwei Drittel der Teilnehmer (67 %) war eine eigenständige rhythmologische Abteilung vorhanden. Ein Großteil der Kliniken der Teilnehmer verfügte über eine invasive Elektrophysiologie (92 %) sowie über eine operative Devicetherapie (94 %).

**Figure 1:**
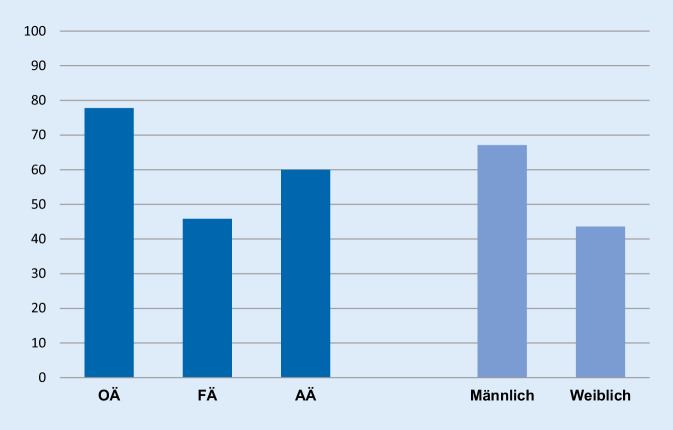

Mehr als die Hälfte der Teilnehmer (53 %) gaben an, mit ihrer kardiologischen Weiterbildung zufrieden zu sein. Im Detail äußerten sich insbesondere Oberärzte und männliche Teilnehmer zufriedener mit ihrer kardiologischen Weiterbildung (Abb. 1).

Insgesamt 66 Teilnehmer (50 %) bewerteten den rhythmologischen Anteil an ihrer Weiterbildung als zu gering und sahen keine Möglichkeit einer invasiven elektrophysiologischen Ausbildung in ihrer Klinik.

Der durchschnittliche Beginn einer rhythmologischen Ausbildung lag in den Kliniken der Teilnehmer zwischen dem 5. und 6. kardiologischen Weiterbildungsjahr. 72 Teilnehmer (55 %) gaben einen relevanten Konkurrenzdruck für ihre invasive Ausbildung an, wobei 79 Teilnehmer (60 %) die Anzahl an invasiven Prozeduren für eine adäquate Ausbildung als zu gering einschätzten.

Von der DGK organisierte rhythmologische Fortbildungsveranstaltungen waren für 31 Teilnehmer (24 %) unbekannt, wurden aber von bereits absolvierten Teilnehmern überwiegend positiv bewertet (70 %). Das onlinebasierte E‑Learning Fortbildungsangebot „ESCeL“ der ESC war 67 Teilnehmern (51 %) nicht bekannt. Fellowship-Programme waren fast der Hälfte der Teilnehmer (48 %) nicht bekannt, wurden aber von den Absolventen ebenfalls sehr positiv bewertet (93 %).

Insgesamt 118 Teilnehmer (90 %) nutzten die Freitextoption, um Verbesserungsansätze zu formulieren. Folgende Optimierungsvorschläge wurden mehrfach genannt:

1. Einführung einer generellen rhythmologischen Rotation,
2. gesteigerter Zugang zu Prozeduren um invasive Fähigkeiten zu erlernen (falls nötig auch klinikübergreifend),
3. intensivierte Werbung für Fortbildungsveranstaltungen und Fellowships,
4. Zunahme onlinebasierter Fortbildungsveranstaltungen.

## Diskussion

Diese Umfrage bietet einen Einblick in die rhythmologische Aus- und Weiterbildung in Deutschland und reflektiert deren Stellenwert und die Zufriedenheit aus Sicht junger Kardiologinnen und Kardiologen.

Die wichtigsten Ergebnisse unserer Umfrage sind:

1. Die Mehrzahl junger Kardiologinnen und Kardiologen ist mit der kardiologischen Weiterbildung sehr zufrieden, wünscht sich allerdings einen größeren Anteil rhythmologischer Inhalte in ihrer Ausbildung.
2. Die vorhandenen rhythmologischen Fortbildungsmöglichkeiten werden überwiegend positiv bewertet, jedoch waren diese auch für einen relevanten Anteil der Teilnehmer unbekannt.
3. Optimierungsansätze werden insbesondere in der Ausweitung rhythmologischer Rotationen sowie in einem intensivierten Angebot an Fortbildungsveranstaltungen gesehen.

Die kardiovaskuläre Medizin und insbesondere die Rhythmologie haben sich durch Wissenszuwachs und durch technologischen Fortschritt innerhalb der letzten Jahre rasant weiterentwickelt. Infolgedessen hat die Projektgruppe Aus‑, Weiter- und Fortbildung der Kommission für Klinische Kardiologie in Zusammenarbeit mit der Arbeitsgruppe Rhythmologie der DGK das „Curriculum Spezielle Rhythmologie“ als eine Qualitätssicherungsmaßnahme sowie als Leitfaden zur Vermittlung von rhythmologischen Lerninhalten und Lernzielen entworfen [5]. Seither erfolgt die Zusatzqualifikation „Spezielle Rhythmologie“ in etwa 200 zertifizierten Ausbildungsstätten in Deutschland.

Eine im Jahr 2010 erstmalig durchgeführte und 2018 aktualisierte Umfrage an deutschen rhythmologischen Zentren verglich die Entwicklungen der Infrastruktur, der Ausbildungsstruktur sowie der Anzahl und technischen Aspekte von Ablationsprozeduren innerhalb der interventionellen Elektrophysiologie zwischen 2010 und 2015 in Deutschland [6, 7]. Diese Umfrage zeigte eine zunehmende elektrophysiologische Spezialisierung mit einem deutlichen Anstieg (1,5-fach) jährlicher elektrophysiologischer Prozeduren (2015: 59.033 vs. 2010: 40.735). Trotz der Zunahme vor allem komplexer elektrophysiologischer Prozeduren (Vorhofflimmerablation: 2‑fach, VT-Ablation: 1,4-fach) blieb die Anzahl elektrophysiologischer Ausbildungspositionen zwischen 2010 und 2015 nahezu konstant. Hierbei erfüllte maximal ein Drittel der teilnehmenden Zentren die empfohlenen Anforderungen der DGK für eine zertifizierte Ausbildungsstätte.

Unsere Umfrage unterstreicht das große Interesse junger Kardiologinnen und Kardiologen an einer rhythmologischen Aus- und Weiterbildung. Aufgrund eines zunehmenden Bedarfs an qualifizierten Rhythmologen/innen ist eine der Grundvoraussetzungen die Ausweitung strukturierter rhythmologischer Ausbildungspositionen. Obwohl 93 % der Kliniken der Teilnehmer rhythmologische Prozeduren anbieten, gaben 66 Teilnehmer (50 %) an, keine Möglichkeit einer strukturierten invasiven Ausbildung zu erhalten und würden, falls möglich, Rotationen in externe Kliniken begrüßen bzw. wahrnehmen.

Die DGK-Akademie bietet u. a. 4 rhythmologische Sachkundekurse (Herzschrittmachertherapie; ICD-Therapie; CRT-Therapie; Elektrophysiologie) und den Intensivkurs EKG-EPU als strukturierte Fortbildungsveranstaltungen an. Diese Kurse wurden von den Teilnehmern der Umfrage, sofern bereits absolviert, sehr positiv bewertet.

Das onlinebasierte E‑Learning-Fortbildungsangebot „ESCeL“ der ESC sowie verschiedene Fellowship-Programme wurden in der Umfrage ebenfalls positiv bewertet, allerdings waren auch diese einem relevanten Anteil der Teilnehmer nicht bekannt.

Da alle Teilnehmer der Umfrage DGK-Mitglieder waren, erhalten diese via E‑Mail regelmäßige Fortbildungsangebote der DGK über die DGK-, AGEP-, und Young DGK-Newsletter. Dies erklärt möglichweise den erhöhten Bekanntheitsgrad DGK-organisierter Fortbildungsveranstaltungen gegenüber dem onlinebasierten Fortbildungsangebot „ESCeL“ der ESC und der Fellowship-Programme (76 % vs. 30 %).

Die Social-Media-Plattform Twitter hat sich innerhalb der kardiologischen Community in den letzten Jahren rasant entwickelt. Durch eine (inter)nationale Vernetzung bietet Twitter eine ideale Weiterbildungsplattform mit Austausch fachlicher und wissenschaftlicher Inhalte [8].

Ein zusätzliches onlinebasiertes interaktives Fortbildungsangebot ist die amerikanische „EP Fellows Conference“, das u. a. über Twitter von Nishant Verma koordiniert wird und auf der Plattform YouTube „on demand“ zur Verfügung gestellt wird. Ein solches Format könnte auch für die rhythmologische Aus- und Weiterbildung in Deutschland nicht nur im Kontext der COVID-19-Pandemie eine spannende zukünftige onlinebasierte Alternative darstellen.

## Fazit für die Praxis

- Die Umfrage unterstreicht das Interesse junger Kardiologinnen und Kardiologen an einer strukturierten, intensivierten und verbesserten rhythmologischen Ausbildung innerhalb ihrer kardiologischen Weiterbildung.
- Onlinebasierte Fortbildungsangebote bieten nicht nur im Kontext der COVID-19-Pandemie eine interessante Alternative zu bereits etablierten Fortbildungsangeboten.

## Caption Electronic Supplementary Material




