# Supplementary material for: Current status of cardiac electrophysiology training in Germany
Source: Herzschrittmacherther Elektrophysiol. 2020 Sep 1;31(4):341–4. [Article in German] doi: 10.1007/s00399-020-00717-4 (PMC7462106; doi:10.1007/s00399-020-00717-4)
Supplement: Supplementary file 1 [file 399_2020_717_MOESM1_ESM.pdf]

Nationales Survey zur kardiologischen Ausbildung in Deutschland

Liebe Kolleginnen und Kollegen,

für ein besseres Verständnis der kardiologischen Ausbildung haben wir diese Umfrage entworfen. Die Bearbeitung dauert nur ca. 5 Minuten und ist freiwillig und anonym.

Herzlichen Dank für die Teilnahme!

Dr. med. K. Willy Dr. med. P. Müller PD Dr. David Duncker

1. Was ist Ihr Geschlecht?

- ◯ Weiblich
- ◯ Männlich
- ◯ Divers

2. In welchem Jahr sind Sie geboren? (Geben Sie das 4-stellige Geburtsjahr an, z. B. 1976)

3. In welchem Ausbildungsstand befinden Sie sich?

- ◯ Medizinstudent/in
- ◯ Assistenzarzt/ärztin im 1.-3. Weiterbildungsjahr
- ◯ Assistenzarzt/ärztin im 4.-6. Weiterbildungsjahr
- ◯ Facharzt/Fachärztin
- ◯ Oberarzt/ärztin

4. Wo sind Sie überwiegend beschäftigt?

- ◯ Universitätsklinik
- ◯ Kirchliches Krankenhaus
- ◯ Krankenhaus in öffentlicher Trägerschaft
- ◯ Krankenhaus in privater Trägerschaft
- ◯ Kardiologische Praxis

5. Hat Ihre Klinik eine eigenständige rhythmologische Abteilung?

- ◯ Ja
- ◯ Nein

6. Welches kardiologische Leistungsspektrum bietet Ihre Klinik? (Mehrfachnennung möglich)

- ☐ Kardiales MRT mit ärztlicher kardiologischer Beteiligung
- ☐ Kardiales CT mit ärztlicher kardiologischer Beteiligung
- ☐ Sportkardiologie
- ☐ Erwachsene mit angeborenen Herzerkrankungen
- ☐ Kinderkardiologie
- ☐ Spezialisierte Kompetenz in der Herzinsuffizienz
- ☐ Koronarangiographie/PCI
- ☐ Interventionelle Herzklappentherapie
- ☐ Invasive Elektrophysiologie
- ☐ Operative Devicetherapie

7. Nach welchem Prinzip erfolgen die Rotationen in Ihrer Klinik Ihrer Meinung nach am ehesten?

- ◯ Zufällig
- ◯ Strukturiert

- ◯ Leistungsorientiert (z.B. Honorierung guter Publikationsleistungen)

8. Wie zufrieden sind Sie mit Ihrer kardiologischen Gesamtweiterbildung insgesamt?

- ◯ Sehr zufrieden
- ◯ Eher zufrieden
- ◯ Weder zufrieden noch unzufrieden
- ◯ Eher unzufrieden
- ◯ Sehr unzufrieden

9. Die rhythmologische Ausbildung in Ihrer Klinik nimmt Ihrer Meinung nach einen angemessenen Stellenwert ein?

- ◯ Stimme voll zu
- ◯ Stimme eher zu
- ◯ Kann ich nicht genau sagen
- ◯ Stimme eher nicht zu
- ◯ Stimmer gar nicht zu

10. Welche Aspekte beinhaltet die rhythmologische Ausbildung in Ihrer Klinik? (Mehrfachnennung möglich)

- ☐ Betreuung rhythmologischer stationärer Patienten
- ☐ Patientenversorgung in einer rhythmologischen Ambulanz
- ☐ Strukturierte EKG-Befundung
- ☐ Langzeit-EKG-Befundung
- ☐ Medikamentöse Differentialtherapie von Arrhythmien
- ☐ Indikationsstellung zur Schrittmacher-und ICD-Therapie
- ☐ Indikationsstellung zur invasiven elektrophysiologischen Untersuchung
- ☐ Implantation von Ereignisrekordern
- ☐ Implantation von Schrittmachern und ICDs
- ☐

11. In meiner Facharztausbildung zum Kardiologen/zur Kardiologin kann ich zu einem geeigneten Zeitpunkt in rhythmologische Funktionsbereiche rotieren.

- ○ Stimme voll zu
- ○ Stimme eher zu
- ○ Kann ich nicht genau sagen
- ○ Stimme eher nicht zu
- ○ Stimme gar nicht zu

12. Falls Ihre Klinik eine Zusatzqualifikation für "Spezielle Rhythmologie" anbietet – in welchem Weiterbildungsjahr beginnt der/die „Funktions-Assistenzarzt/ärztin Rhythmologie" mit der Ausbildung?

1 Jahr 6

○

13. Folgende Punkte treffen auf meine rhythmologische Ausbildung zu:

- ☐ Ausreichendes Teaching bezüglich rhythmologischer Differential-und EKG-Diagnostik
- ☐ Ausreichende Anzahl an Prozeduren ist für eine adäquate Ausbildung/Erreichung der Zusatzbezeichnungen gegeben
- ☐ Hohe Konkurrenz innerhalb der Gruppe der Assistenz- und Fachärzte um invasive rhythmologische Ausbildung
- ☐ Ausreichende interne Fortbildungsveranstaltunge n gegeben
- ☐ Ausreichend Freiraum zur selbstständigen Fort-und Weiterbildung gegeben

14. In meiner Klinik ist es mir möglich, in der Devicetherapie ausgebildet zuwerden

- ◯ Stimme voll zu
- ◯ Stimme eher zu
- ◯ Kann ich nicht genau sagen
- ◯ Stimme eher nicht zu
- ◯ Stimme gar nicht zu

15. In meiner Klinik ist es mir möglich in der invasiven Elektrophysiologie ausgebildet zu werden

- ◯ Stimme voll zu
- ◯ Stimme eher zu
- ◯ Kann ich nicht genau sagen
- ◯ Stimme eher nicht zu
- ◯ Stimme gar nicht zu

16. Mir stehen meiner Meinung nach ausreichend externe Fortbildungsmöglichkeiten zum Thema Rhythmologie zur Verfügung.

- ◯ Stimme voll zu
- ◯ Stimme eher zu
- ◯ Kann ich nicht genau sagen
- ◯ Stimme eher nicht zu
- ◯ Stimme gar nicht zu

17. Im Folgenden zeigen wir Ihnen einige organisierte Fortbildungsprogramme und möchten wissen, inwiefern Ihnen diese bekannt sind bzw. bereits genutzt worden.

1. Sachkundekurse (HSM/ICD/CRT, EP)

- ◯ Ist mir nicht bekannt
- ◯ Ist mir bekannt, allerdings aktuell nicht interessant für mich
- ◯ Ist mir bekannt, kann mir eine Teilnahme vorstellen
- ◯ Eine Teilnahme ist bereits geplant
- ◯ Bereits teilgenommen, war nicht zufrieden
- ◯ Bereits teilgenommen, war zufrieden

- Würde gerne teilnehmen, allerdings aktuell mit dem beruflichen Alltag nicht vereinbar

18. 2. Intensivkurs Interventionelle Elektrophysiologie

- Ist mir nicht bekannt
- Ist mir bekannt, allerdings aktuell nicht interessant für mich
- Ist mir bekannt, kann mir eine Teilnahme vorstellen
- Würde gerne teilnehmen, allerdings aktuell mit meinem beruflichen Alltag nicht vereinbar
- Eine Teilnahme ist bereits geplant
- Bereits teilgenommen, war nicht zufrieden
- Bereits teilgenommen, war zufrieden

19. 3. Intensivkurs EKG/EPU

- Ist mir nicht bekannt
- Ist mir bekannt, allerdings aktuell nicht interessant für mich
- Ist mir bekannt, kann mir eine Teilnahme vorstellen
- Würde gerne teilnehmen, allerdings aktuell mit meinem beruflichen Alltag nicht vereinbar
- Eine Teilnahme ist bereits geplant
- Bereits teilgenommen, war nicht zufrieden
- Bereits teilgenommen, war zufrieden

20. 4. E-Learning Plattform der EHRA (ESCeL)

- Ist mir nicht bekannt
- Teilnahme bereits geplant

Ist mir bekannt, allerdings aktuell nicht interessant für mich

- ◯ Ist mir bekannt, kann mir eine Teilnahme vorstellen
- ◯ Würde gerne teilnehmen, allerdings aktuell mit meinem beruflichen Alltag nicht vereinbar

Bereits teilgenommen, war nicht zufrieden

- ◯ Bereits teilgenommen, war zufrieden

21. 5. Fellowship Programme

- ◯ Ist mir nicht bekannt
- ◯ Ist mir bekannt, allerdings aktuell nicht interessant für mich
- ◯ Ist mir bekannt, kann mir eine Teilnahme vorstellen
- ◯ Würde gerne teilnehmen, allerdings aktuell mit meinem beruflichen Alltag nicht vereinbar
- ◯ Teilnahme bereits geplant
- ◯ Bereits teilgenommen, war nicht zufrieden
- ◯ Bereits teilgenommen, war zufrieden

22. Insbesondere in diesen Bereichen besteht meiner Meinung nach Optimierungspotenzial für die Ausbildung in der Rhythmologie:

Von Seiten meiner Klinik:

Von extern:

Fertig
